# Crossley Sanatorium, Delamere Forest, near Frodsham, Cheshire

**Published:** 1905-08-12

**Authors:** 


### CROSSLEY SANATORIUM, DELAMERE FOREST, NEAR FRODSHAM, CHESHIRE.

This splendid sanatorium for consumption owes its existence to the munificence of Mr. Crossley, of Manchester, who erected it at a cost of £70,000; and the indebtedness of Manchester does not end there, for another member of the family presented the institution with the furniture. Three years were occupied in building, and the architect was Mr. W. Hardisty, of Manchester; but many of the details were carried out under the superintendence of the medical director, Dr. Lloyd Smith, and these details show a singularly accurate knowledge of the requirements of a sanatorium of this kind. The site of the building is an extensive one running to about 66 acres. It is about four miles from the Frodsham Station, on the outskirts of Delamere Forest, high above the sea-level, and commands extensive and beautiful views, the Welsh hills being well in evidence towards the west. Surely an ideal spot for a hospital of any kind. Scattered about the grounds are many shelters and kiosks, but these are all for day-use only.

The main front of the sanatorium faces south-by-east, or nearly so. As we hope to publish the plans in some future issue, we need not do more at present than state that the design is extremely simple; that the public rooms are large and beautiful; the corridors wide and well-lighted; and that the cross ventilation of the small bedrooms and dormitories is efficiently carried out—better, we think, than in any institution of its kind we have yet visited. The accommodation is for 90 beds, and of this total, 60 are intended for patients who are received free of payments, and 30 for patients who pay from two guineas to three guineas a week. The free patients all pass through one of the Manchester hospitals. At that institution a selection is made, and only those in the first stage of the disease are sent to Delamere. On the other hand, paying patients are not subjected to this selection, and hence there will be available in course of time a series of statistics illustrating the relative curableness of phthisis in the early and subsequent stages of the disease. So far the results of the treatment carried out at Delamere have been decidedly favourable, but, as the sanatorium has only been opened a few months, no statistics are as yet published. No special line of medical treatment is followed here. It is almost entirely dietetic and hygienic. Three good meals are given, and in addition there is an early cup of cocoa or hot milk. Patients are instructed to eat all that is given to them, especially red meats, bacon, eggs, milk, and butter. No alcoholic drink forms any part of the regular diet.

Even a short visit to this sanatorium would convince any one that there is a very distinct, palpable medical element permeating the whole institution. It is emphatically a hospital. In addition to the medical director there are two resident physicians, and as there are three floors to the building, each medical officer takes in turn one floor as his special province. By this arrangement all the physicians become thoroughly familiar with all the patients. Every patient after going to bed is seen by one of the medical staff, and there is an early morning visit at which the patient receives instructions for the day. The smallest points of administration have been considered by Dr. Lloyd Smith, and are carried into practice by his medical colleagues, nurses and staff generally. Charts are kept for every patient, and these show the name, age, height, temperature, pulse, &c., as well as the name of the medical officer under whose immediate care the patient may be; and in addition to this every patient carries about with him a "daily routine card," on which are given full instructions for the day. These cards are of three colours—red for those patients who have "rest" prescribed for them; green for those who have "rest and exercise"; and white for those who have "exercise." The spittoons in use are coloured green for the women and white for the men. These spittoons are collected regularly, the contents are destroyed, and the vessels sterilised. To simplify collection and distribution a very ingenious wagon has been invented by Dr. Smith. This wagon is arranged in sections; each floor of the sanatorium has its own section, but any one can be got at without disturbing the others.

The operating theatre may be taken as a model of what such an adjunct should be in a sanatorium. The walls are of Portland cement colour. The instrument cupboards are of phosphor-bronze and glass, and were made by Arnold and Co. The vessels used are of two colours, green and red. When an operation or examination is being made, the green glasses contain the clean instruments and the red glasses contain those which have been used. Every patient on admission, and as often as necessary afterwards, has a thorough examination made of his throat, nose, and teeth, so that the theatre is in frequent use. It is Dr. Smith's opinion that phthisis pulmonalis is not necessarily associated with a high bodily temperature, but that the high temperature so often found is due to some intercurrent affection, hence the urgent necessity for frequent and careful examination of all parts likely to be implicated.

The bathing arrangements are unusually good. The sitz, shower, spray, and douche baths can all be controlled by an attendant from one stand placed near the middle of the floor. This stand was invented by the medical director, and carried out by Doulton and Co. There is an electric apparatus department, and the installation and general fitting up of this room seem perfect.

The kitchen and scullery contain the most recent appliances for the preparation and cooking of food; and after the plates have been used they are washed by a machine which thoroughly cleans them in a few seconds.

The post mortem room, mortuary, and sputa-examination room form a separate block. Here, too, all the arrangements and appliances are exceptionally good. The windows of these rooms are glazed with red-coloured glass.

